# Utilizing collimated aperture with proton pencil beam scanning (PBS) for stereotactic radiotherapy

**DOI:** 10.1002/acm2.14362

**Published:** 2024-04-26

**Authors:** Chen‐Yu Chou, Tsung‐Shiau Tsai, Hsiao‐Chieh Huang, Chun‐Chieh Wang, Shen‐Hao Lee, Shih‐Ming Hsu

**Affiliations:** ^1^ Department of Radiation Oncology Linkou Chang Gung Memorial Hospital Taoyuan City Taiwan (R.O.C); ^2^ Department of Biomedical Imaging and Radiological Sciences National Yang Ming Chiao Tung University Taipei City Taiwan (R.O.C)

**Keywords:** aperture, IMPT, PBS, PSRS, radiosurgery

## Abstract

**Purpose:**

Proton stereotactic radiosurgery (PSRS) has emerged as an innovative proton therapy modality aimed at achieving precise dose delivery with minimal impact on healthy tissues. This study explores the dosimetric outcomes of PSRS in comparison to traditional intensity‐modulated proton therapy (IMPT) by focusing on cases with small target volumes. A custom‐made aperture system designed for proton therapy, specifically tailored to small target volumes, was developed and implemented for this investigation.

**Methods:**

A prerequisite mechanical validation through an isocentricity test precedes dosimetric assessments, ensuring the seamless integration of mechanical and dosimetry analyses. Five patients were enrolled in the study, including two with choroid melanoma and three with arteriovenous malformations (AVM). Two treatment plans were meticulously executed for each patient, one utilizing a collimated aperture and the other without. Both plans were subjected to robust optimization, maintaining identical beam arrangements and consistent optimization parameters to account for setup errors of 2 mm and range uncertainties of 3.5%. Plan evaluation metrics encompassing the Heterogeneity Index (HI), Paddick Conformity Index (CI_Paddick_), Gradient Index (GI), and the R50% index to evaluate alterations in low‐dose volume distribution.

**Results:**

The comparative analysis between PSRS and traditional PBS treatment revealed no significant differences in plan outcomes, with both modalities demonstrating comparable target coverage. However, collimated apertures resulted in discernible improvements in dose conformity, dose fall‐off, and reduced low‐dose volume.

**Conclusions:**

This study underscores the advantageous impact of the aperture system on proton therapy, particularly in cases involving small target volumes.

## 1 INTRODUCTION

Intracranial tumors have long posed significant challenges due to their proximity to critical organs within the brain. Surgical interventions are often complex when these tumors are in close proximity to organs at risk (OAR). Stereotactic radiotherapy has emerged as a non‐invasive alternative to surgical treatments for intracranial lesions.^1^, ^2^ As per the AAPM Task Group 101 report,^3^ radiotherapy includes stereotactic radiosurgery (SRS), which delivers a high dose in a single fraction, and stereotactic radiotherapy (SRT), which involves multi‐session radiosurgery (typically two to five sessions) with larger doses (≥5 Gy per fraction). Compared to conventional external beam radiotherapy (EBRT), which administers 1.8–2.0 Gy per fraction over 5−6 weeks, SRS/SRT offers shorter treatment durations and an improved quality of life for patients.^4^

While photon therapy can achieve excellent dose conformity and uniformity, it still presents challenges, notably in the form of a large exit dose region, leading to unnecessary exposure of normal tissues. Proton beam therapy leverages its unique physical properties, with the Bragg peak being its standout feature. When a proton beam reaches a certain depth, it releases its energy entirely, sparing the OAR behind the tumor from radiation. A uniform dose distribution along the irradiation axis can be achieved by employing precise modulation.^5^ Alternatively, pencil beam scanning (PBS) can create a dose distribution tailored to the tumor's shape. These properties make proton therapy superior in dose distribution, significantly reducing normal tissue irradiation while allowing higher radiation doses to be delivered to the tumor.^6^

Ensuring a conformal dose distribution and rapid dose fall‐off beyond the tumor is imperative to treat small lesions effectively. However, proton beams may create lateral penumbras due to multiple Coulomb scattering.^7^, ^8^ These penumbras can be attributed to scattering from the treatment nozzle or a relatively larger spot size when employing low‐energy proton beams. A similar situation arises when using a snout degrader, also called a range shifter.^9^ Combining PBS technology with a collimated aperture offers an effective solution for minimizing penumbra size.^10^

The utilization of PBS in conjunction with an aperture represents an effective approach for achieving a rapid lateral dose reduction, as outlined in the references Smith et al.^11^ and Suit et al.^12^ This fusion of PBS with an aperture can be executed through several methods, encompassing static apertures, multi‐leaf collimators, and dynamic collimators, all of which can operate seamlessly alongside PBS, as elucidated in references Verhey et al.^13^ and Vilches‐Freixas et al.^14^ When the aperture is strategically positioned between the range shifter and the patient, it substantially enhances the lateral dose gradients, resulting in an approximate 20% augmentation, as detailed in the reference Yoo et al.^15^

Confirming the advantages of employing a collimated aperture, we have developed an aperture system in collaboration with Sumitomo Heavy Industry, Ltd. (SHI) specifically for treating small lesions at Linkou Chang Gung Memorial Hospital (CGMH).

## 2 MATERIAL AND METHODS

### 2.1 Proton beam system equipment

Proton irradiation was administered utilizing the P235 cyclotron manufactured by Sumitomo Heavy Industries (SHI) and conducted in line‐scanning proton PBS mode. The SHI proton delivery system facilitated the acceleration of proton beams to a consistent energy level of 230 MeV, with the capability for continuous modulation down to 70 MeV at the termination point of the cyclotron, thanks to the deployment of an energy degrader. In‐air proton dose distributions manifested as Gaussian profiles, characterized by sigma values at the isocenter that spanned the spectrum from approximately 7 to 18 mm for proton beams of 230 MeV and 70 MeV, respectively. The maximum field size attainable measured 30 cm by 40 cm. The proton beam's depth range within a water medium exhibited variations ranging from 38 to 324 mm. To accommodate the treatment of superficial lesions and ensure adequate dose coverage, a snout degrader with a water equivalent thickness (WET) measuring 40.7 mm could be judiciously employed.

### 2.2 Proton treatment planning system

The Eclipse treatment planning system, version 13.7 by Varian Medical Systems, was utilized in conjunction with the line‐scanning modality of the SHI proton therapy system. The optimization algorithm employed within this study was the Nonlinear Universal Proton Optimizer, denoted as ver. 13.7.21. Given the intricate nature of proton stereotactic radiosurgery (PSRS) and its imperative demand for high‐dose treatment planning within small target volumes, an innovative algorithm named PCS_PBSRS (Proton Convolution Superposition ver. 13.7.21) was conceived and implemented. This algorithm facilitated a remarkable increase in the maximum dose rate, elevating it from 215.487 MU/s to an impressive 1045 MU/s while preserving the minimum dose rate limit of the treatment planning system at 16.8 MU/s. The available field sizes spanned from 1 to 6 cm, and the layer spacing exhibited a range from 3.5 to 2.0 sigma, complemented by a fine dose calculation resolution of 5 mm spot spacing.

### 2.3 Design of the collimated aperture device

The collimating approach adopted in this study entails using a static brass aperture. This choice arises from considerations of the intrinsic design of the proton therapy system's nozzle. Opting for the static brass aperture circumvents the necessity for substantial renovations and upgrades to the entire nozzle system, potentially preventing operational downtime. Therefore, our study proposes the development of an accessory holder capable of accommodating the static brass aperture or a snout degrader without disrupting routine treatment protocols. This static aperture design offers practical advantages in system compatibility, ease of implementation, and minimal interference with current treatment workflows.

Following thorough communication and research collaboration with SHI, we designed and manufactured a collimated aperture system dedicated to PSRS and stereotactic radiotherapy (PSRT). The aperture system comprises a brass block with a diameter of 9 cm installed upstream. In the middle section, there is a 4 cm physically thick snout degrader used to pull back the proton range to the surface and treat superficial tumors. A 6 cm thick patient‐specific collimated brass aperture is placed downstream to reduce the penumbra region. The available aperture sizes for clinical treatment range from 1 to 6 cm.

**FIGURE 1:**
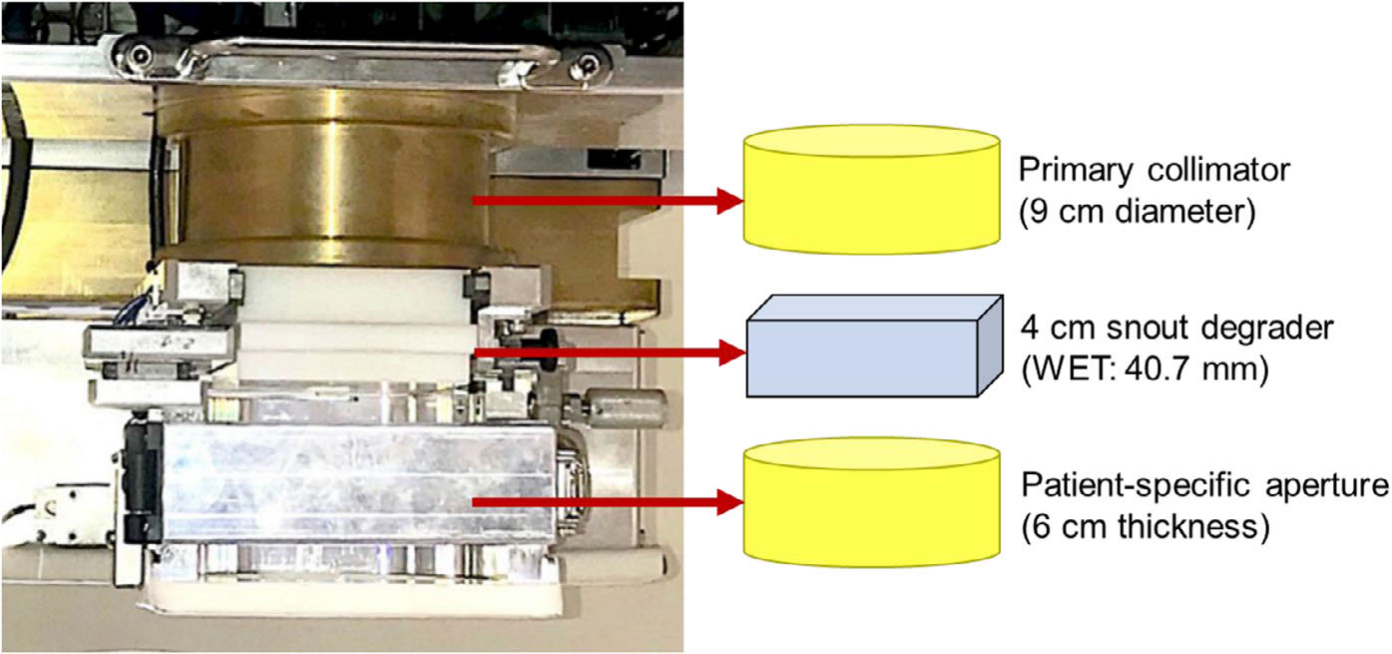
The components of the collimated aperture device. Upstream is a primary collimator with a diameter of 9 cm. Patient‐specific aperture downstream with snout degrader located in the middle.

Figure 1 illustrates the geometric structure of the aperture device.

### 2.4 Mechanical performance validation

**FIGURE 2:**
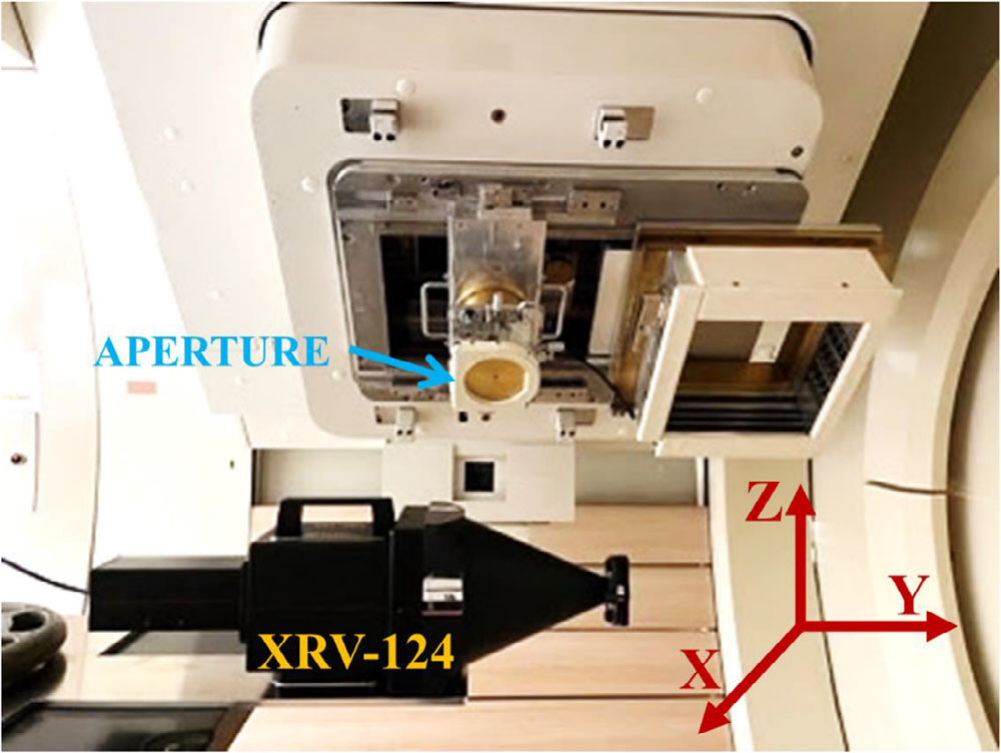
Schematic diagram used for measurement by XRV‐124. The X‐axis is L‐R, the Y‐axis is S‐I, and the Z‐axis is A‐P.

Upon the completion of aperture device manufacturing, an essential precursor to dosimetry evaluation is the validation of mechanical performance, notably through an isocentricity test. Within this study, we conducted feasibility tests using an XRV‐124 scintillation detector (Logos Systems Int'l, Scotts Valley, California) to measure the consistency of x‐ray systems with uniformly scanned proton beams,^16^, ^17^, ^18^ as shown in Figure 2. The alignment procedure of the XRV‐124 scintillation detector entails utilizing an x‐ray imaging system to position it accurately within the treatment room. This involves initiating a gantry start shot with the x‐ray imaging system and iteratively verifying the positioning of the detector. The verification is carried out using an orthogonal x‐ray imaging system until minimal residual correction vectors are achieved.

To assess the position of the beam spot, the XRV‐124 detector is subjected to a single pencil beam at the center from various gantry angles. This approach ensures comprehensive coverage and accurate assessment of the beam spot position.

### 2.5 Integration in treatment planning

When using a collimated aperture, it is crucial to determine the optimal margin range. A margin that is too small may result in poor dose coverage, while a margin that is too large can compromise the advantages of the aperture. A 6 cm default air gap was set, and both single field optimization (SFO) and multiple field optimization (MFO) were employed, depending on plan complexity. Parameter settings for robust optimization included a 2 mm setup error and a 3.5% calibration curve error.

**FIGURE 3:**
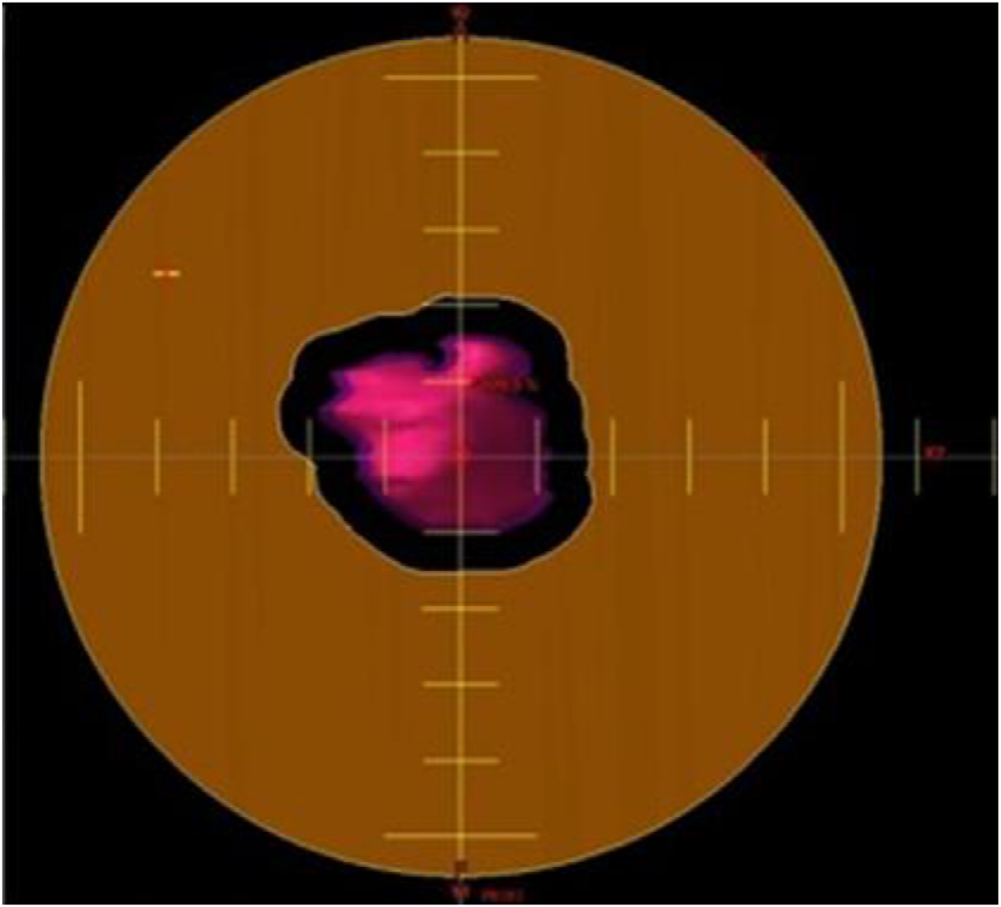
Example of an aperture design derived from the target geometry of a given treatment field.

In the treatment planning process, the aperture size and shape are delineated based on the unique specifications of each treatment field. This entails a planning process similar to traditional 3D RT and passive scattering PT techniques. Illustrated in Figure 3 is an example of an aperture design derived from the target geometry of a given treatment field. Once the aperture size and shape are determined for a specific treatment field, it is important to ensure that the aperture is integrated during the planning optimization phase. This step ensures that the planning outcome accurately reflects the IMPT with aperture insert calculation result, thereby enabling a tailored and precise approach aligned with the personalized requirements of each patient.

### 2.6 Dosimetry evaluation

Five previously treated cases were replanned with the collimated aperture device and compared with the original treatment plans. Table 1 provides an overview of all cases included in this study. To evaluate whether PSRS surpasses traditional PBS regarding plan outcomes, we implement various relevant quality indices during the evaluation process. In assessing whether there is any statistically significant difference among the indices, we conducted a paired *t*‐test for *p*‐value analysis.

**TABLE 1:** Overview of five cases which were included in the current study.

Patient	Diagnosis	Lesion site	Volume [cc]	Aperture size [cm × cm]	Dose [Gy]	Fraction number	Optimization approach	Beam arrangement^a^
#1	Choroid melanoma	Right eye	1.3	2.72 × 2.36	50	5	SFO	G240C0 G330C90
#2	Choroid melanoma	Left eye	0.6	2.79 × 2.36	50	5	SFO	G330C40 G50C30
#3	AVM	Superior vermis	4.8	2.18 × 2.33	18	3	SFO	G250C0 G110C0 G210C90
#4	AVM	Left para‐thalamus Temporal lobe	23.4	2.09 × 2.29	18	3	MFO	G40C0 G120C300 G180C0
#5	AVM	Right corpus callosal splenium	9.3	2.99 × 3.66	18	3	SFO	G180C0 G250C0 G240C90

Abbreviations: AVM, arteriovenous malformation; MFO, multiple field optimization; SFO, single field optimization.

^a^ Beam arrangement: G stands for gantry angle. C stands for couch angle.

The Heterogeneity Index (HI) was used to assess differences in dose distribution between treatment plans, ensuring that plans using the collimated aperture maintained the same plan quality. The HI is given here,

(1) $$HI=\frac{D_{5}}{D_{95}}$$

The utilization of CI_Paddick_ ^19^, ^20^, ^21^ (as advised in ICRU Report 91) was employed to evaluate the conformity of the prescribed dose distribution. The following equation delineates the calculation:

(2) $$CI_{Paddick}=\frac{{\left(TV_{PIV}\right)}^{2}}{TV\times PIV}$$

Here, TV_PIV_ represents the target volume covered by the prescription isodose volume (PIV), and TV denotes the target volume. The reason for not incorporating CI_RTOG_, calculated as the ratio of the reference isodose volume to the target volume, in the study lies in its limitation to adequately account for the spatial overlap degree between the prescribed isodose and the target volume, as well as their respective shapes.^22^ This limitation poses a risk of generating misleading conformity scores, undermining the reliability of the results.

The Gradient Index (GI) is defined as the ratio of the 50% isodose(V_50_) volume to the PIV.^19^

(3) $$GI=\frac{V_{50}}{PIV}$$

This parameter within the realm of plan evaluation serves as a quantifiable metric for assessing the steepness of the dose gradient beyond the target volume. Consequently, the GI assumes a crucial role alongside the conformity index in delineating the quality of a given treatment plan. Particularly within the PSRS plans, the dose fall‐off rate beyond the target volume holds profound significance as an indicator of plan efficacy, notably in predicting potential complications.

Another principal metric for plan evaluation is R50% to evaluate the low‐dose regions.^23^

(4) $$R50\%=\frac{V_{50}}{TV}$$

## 3 RESULTS AND DISCUSSION

### 3.1 Isocentricity verification

The XRV‐124 was utilized to assess the deviation between the proton beam and the image position at the isocenter for different gantry angles. As the gantry undergoes rotation within the X‐Z plane, the dataset inherently captures transversal displacements in the cross‐plane direction along the gantry axis. Conversely, given the perpendicular orientation of the Y‐axis to the gantry rotation plane, the resultant data inherently encompasses longitudinal displacements within the in‐plane direction along the gantry axis.

**FIGURE 4:**
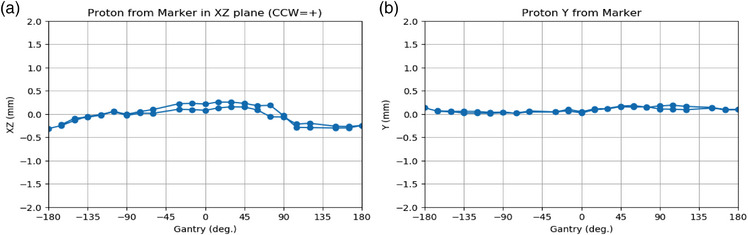
The proton beam position was measured by XRV‐124 at different gantry angles. (a) In the X‐Z plane, the maximum isocenter deviation is 0.34 mm at 180 degrees. (b) The isocenter deviation measured at all gantry angles is all within 0.3 mm in the Y‐axis. Notably, the two connected lines depicted in each plot represent measurements obtained while rotating the gantry in two distinct directions: clockwise and counterclockwise.

The maximum deviation measured was 0.34 mm at 180 degrees in the X‐Z plane. Isocenter deviations measured at all gantry angles were within 0.5 mm, as shown in Figure 4, consistent with clinical use standards. The two connected lines in each dataset depicted in each plot represent measurements obtained while rotating the gantry in two distinct directions: clockwise and counterclockwise. This distinction is paramount due to the structural configuration of the gantry rotating bearing, which may induce deviations that vary between rotation directions. These results support the clinical utility of the collimated aperture system.

### 3.2 Plan analysis

**FIGURE 5:**
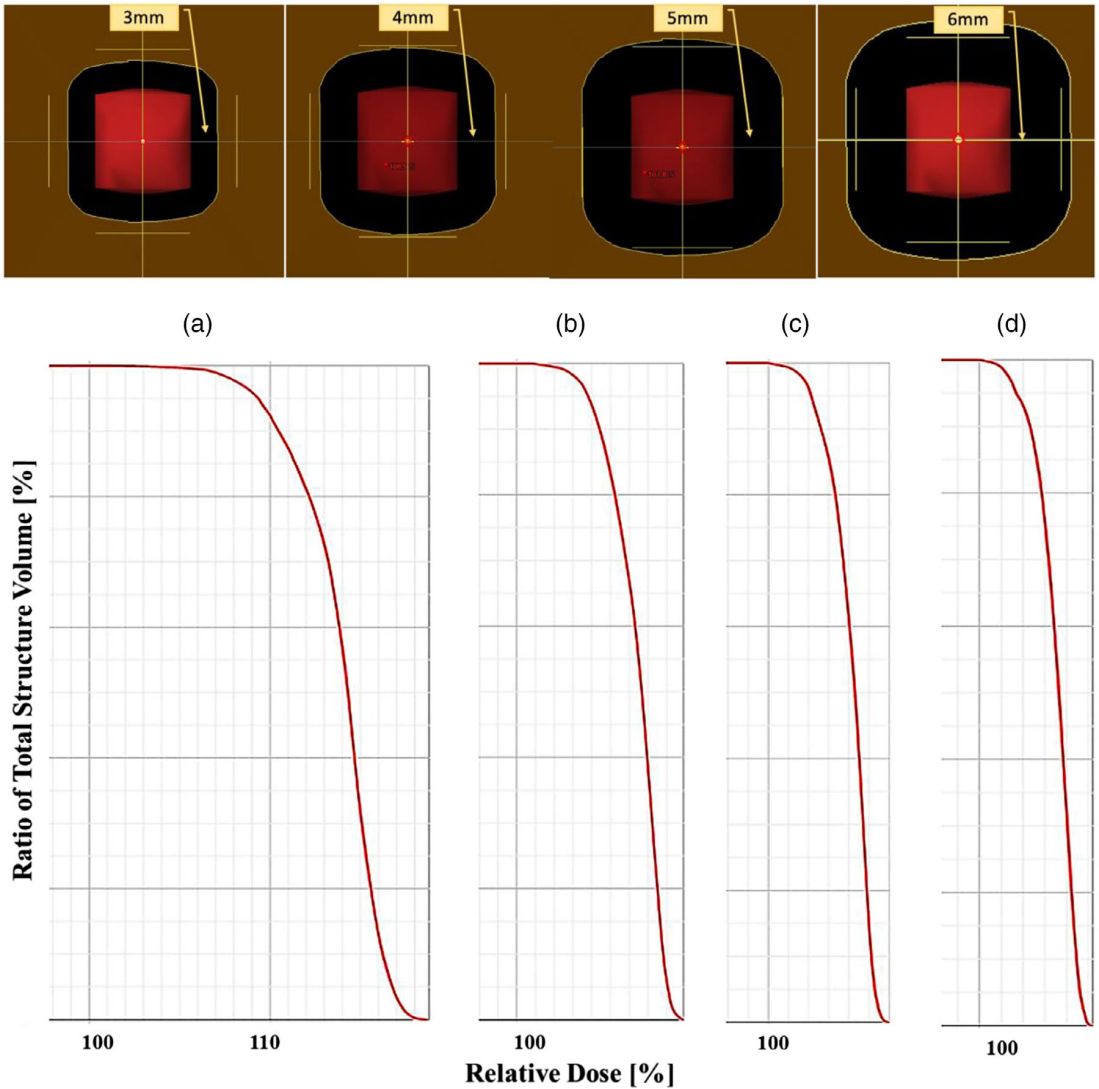
Use a target with a length of 1 cm to match different margins for treatment planning. The dose volume histogram shows the target optimized with (a) 3 mm, (b) 4 mm, (c) 5 mm, and (d) 6 mm margin. The coverage of the target is 100% in all treatment plans.

The choice of margin expansion from the target significantly affects tumor dose coverage. We used different margin sizes (3–6 mm) for tumors of various sizes (1–4 cm), and separate treatment plans were created, as shown in Figure 5. With uniform dose coverage as a consideration, a 5 mm dedicated margin proved effective in achieving good tumor dose coverage. This margin value effectively balances the imperative of maintaining aperture benefits while also considering the potential effects of setup errors on dose distribution.

Five cases were included in this pilot study, encompassing two cases of choroid melanoma (located in the left and right eyes) and three cases of arteriovenous malformation (AVM) (located in the superior vermis, left para‐thalamus temporal lobe, and right corpus callosal splenium). The volumes of these lesions ranged from 0.6 to 23.4 cc. The total therapeutic dose for choroid melanoma was 50 Gy, administered over five treatments, while AVM cases received a total therapeutic dose of 18 Gy, delivered in three treatments. Both SFO and MFO methods were employed.

Table 2 shows no significant differences in HI value between the PBS and PSRS types of treatment plans. However, the employment of the MFO approach has the potential to yield inhomogeneous plan outcomes, as shown in Case 4, owing to the intricate characteristics of target morphology and the demands of plan optimization. Despite the inherent complexity associated with MFO, our clinical findings have demonstrated improvements in tumor coverage and the mitigation of radiation dosage to surrounding critical structures. Nevertheless, under typical circumstances, the utilization of SFO maintains its preference due to its effectiveness in mitigating uncertainties in beam delivery.

**TABLE 2:** Heterogeneity Index (HI) in PBS and PSRS treatment plans.

Patient	D_95, PBS_ [Gy]	D_5, PBS_ [Gy]	D_95, PSRS_ [Gy]	D_5, PSRS_ [Gy]	HI_PBS_	HI_PSRS_
#1	41.23	55.47	42.25	54.51	1.35	1.29
#2	50.04	60.47	48.02	57.72	1.21	1.20
#3	17.22	19.78	17.23	19.39	1.15	1.12
#4	13.37	19.81	13.35	19.74	1.48	1.48
#5	17.99	19.09	18.04	18.92	1.06	1.05
					*p*‐value = 0.05	

*Note*: Heterogeneity Index (HI) = D_5_/D_95._ D5: minimum dose in 5% of the Target volume, indicating maximum dose. D95: minimum dose in 95% of the Target volume, indicating minimum dose.

Table 3 reveals that the conformity index is derived from the calculation approach outlined in Equation 2. A CI value closer to one indicates superior tumor dose conformity. As revealed in Table 3, PSRS plans demonstrated superior outcomes to the PBS plan across the conformity index evaluated with statistical significance for a *p*‐value of 0.01.

**TABLE 3:** Paddick conformal index (CI_Paddick_), GI, and R50% in PBS and PSRS treatment plans.

	CI_Paddick_	CI_Paddick_	GI	GI	R50%	R50%
Patient	PBS	PSRS	PBS	PSRS	PBS	PSRS
#1	0.44	0.49	26.87	9.41	17.78	9.12
#2	0.31	0.49	12.71	6.41	40.66	13.15
#3	0.44	0.62	4.95	4.13	10.79	6.40
#4	0.44	0.50	4.27	2.78	7.18	4.20
#5	0.37	0.49	3.85	3.13	10.13	6.20
*p*‐value	0.01		0.08		0.05	
	$CI_{Paddick}=\frac{{\left(TV_{PIV}\right)}^{2}}{TV\times PIV}$		$GI=\frac{V_{50}}{PIV}$		$R50\%=\frac{V_{50}}{TV}$	

Abbreviations: GI, Gradient Index; PBS, pencil beam scanning; PIV, the prescription isodose volume; PSRS, Proton stereotactic radiosurgery; TV, the target volume; TV_PIV_, the target volume covered by the prescription isodose volume; V_50_, 50% isodose volume.

The GI and R50% values of PSRS in Table 3 show better performance than those of PBS. While statistical significance in GI was not observed, the consistently lower values for PSRS plans are noteworthy. The observation in GI and R50% represents a sharper dose fall‐off and a reduction in the low‐dose region near the target boundary with the aperture's configuration, resulting in lower collateral damage to surrounding tissues or critical organs.

**FIGURE 6:**
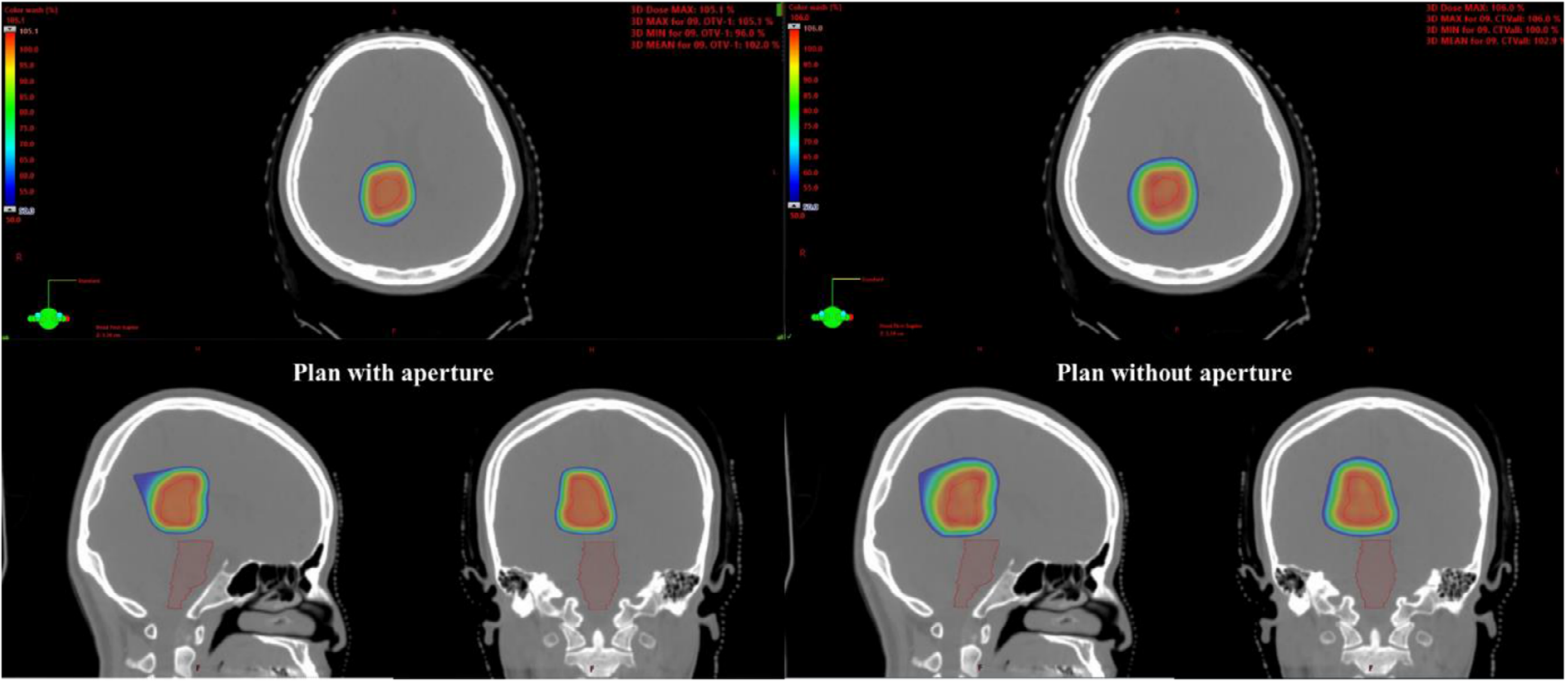
The 50% dose distribution in the arteriovenous malformation (AVM) case was planned with a collimated aperture (left) and an uncollimated plan (right).

**FIGURE 7:**
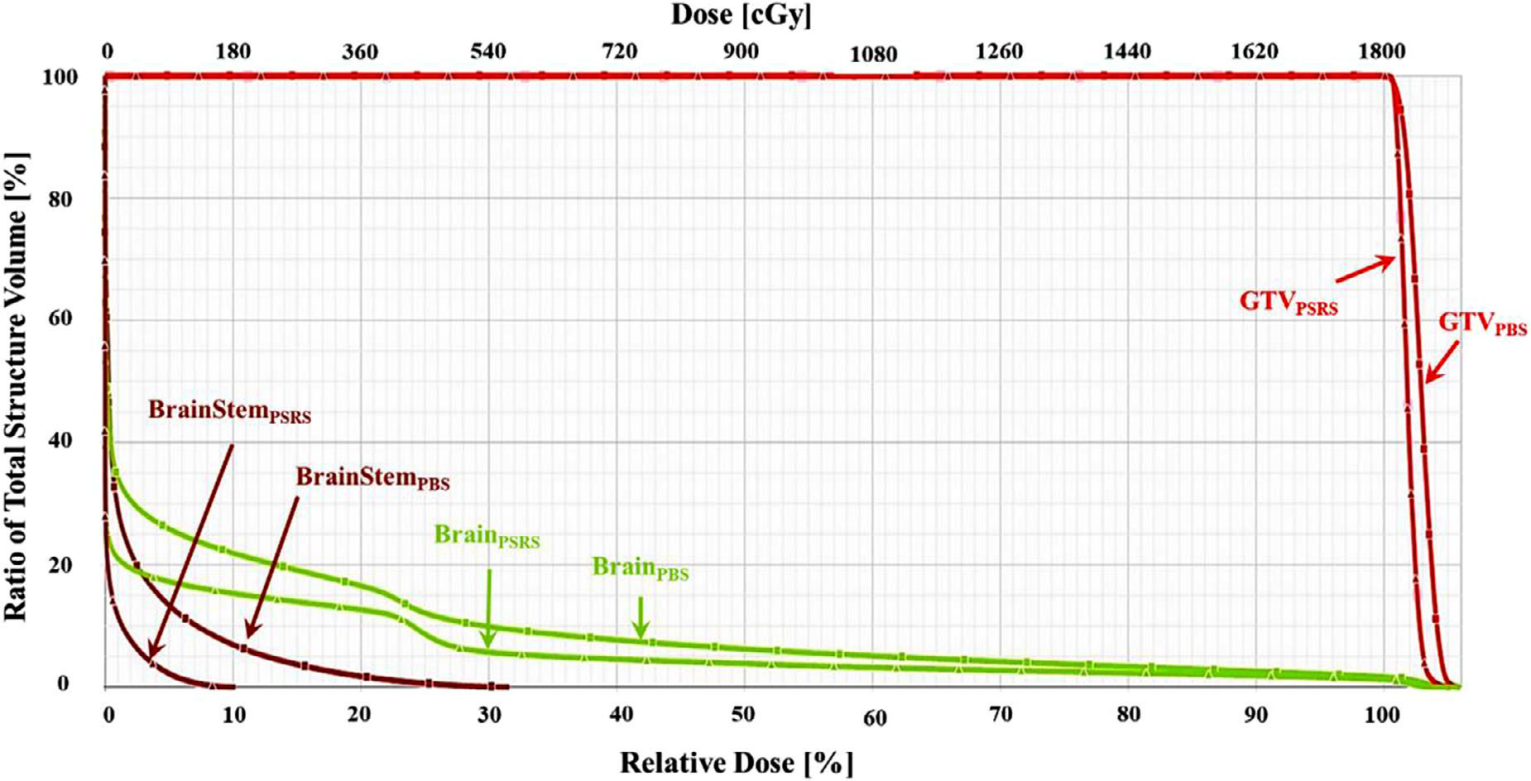
The dose‐volume histogram shows the difference between collimated and uncollimated plans. GTV remains the same coverage. Meanwhile, the brainstem and brain tissue reduce the dose received.

Figures 6 and 7 show the dose distribution and dose‐volume histogram (DVH) for case 5, an AVM case. Figure 6 illustrates a discernible decline in the area receiving 50% of the prescribed dose when utilizing an aperture. In Figure 7, the target maintained full target coverage accompanied by a noteworthy reduction in the maximum dose delivered to the brainstem, decreasing from 31% to 10% with the employment of the aperture plan. Furthermore, the volume of normal brain tissue exposed to irradiation was significantly reduced, offering clinical advantages with the utilization of the aperture.

## 4 CONCLUSION

The development and successful integration of the collimated aperture system into proton therapy for intracranial lesions signify a significant advancement in radiation oncology. Through collaborative efforts and meticulous research, we have engineered a solution that addresses the challenges of treating small lesions near critical organs, thereby enhancing both treatment precision and quality.

The rigorous validation and verification processes have underscored the reliability and accuracy of the collimated aperture system. From mechanical performance tests to dosimetry evaluations, the results consistently demonstrate the system's capability to achieve superior dose conformity and minimize radiation exposure to healthy tissues.

Plan analysis and dosimetry evaluations of treated cases have provided compelling evidence of the collimated aperture system's efficacy. Notably, the system has shown reduced irradiation of critical structures and sharper dose gradients compared to conventional proton therapy techniques. These outcomes translate into tangible clinical benefits, including enhanced treatment efficacy and reduced risk of side effects for patients.

As we prepare to implement the collimated aperture system into clinical practice, our focus remains on ensuring patient safety and treatment efficacy. Future work, including absolute dose measurements and comprehensive dose distribution comparisons, will further validate the system's performance in clinical practice.

In conclusion, the collimated aperture system represents a significant step forward in pursuing more precise and effective treatments for stereotactic radiotherapy. We are poised to improve patient outcomes and push the boundaries of modern radiation oncology by harnessing the power of proton therapy and innovative engineering.

## AUTHOR CONTRIBUTIONS

Chen‐Yu Chou participated in the study design, coordinated the research, carried out treatment plans, collected data, performed data analysis, and drafted the manuscript. Tsung‐Shiau Tsai contributed to data collection. Hsiao‐Chieh Huang and Shen‐Hao Lee contributed to coordinating various parties in this study. Chun‐Chieh Wang provided the cases utilized in the study. Shih‐Ming Hsu offered guidance and reviewed the manuscript. All authors have read and approved the final version of the manuscript.

## CONFLICT OF INTEREST STATEMENT

The authors declare no conflicts of interest.
